# Inhibition of cardiolipin biosynthesis partially suppresses the sensitivity of an *Escherichia coli* mutant lacking OmpC to envelope stress

**DOI:** 10.71150/jm.2507004

**Published:** 2025-11-30

**Authors:** Dae-Beom Ryu, Umji Choi, Gyubin Han, Chang-Ro Lee

**Affiliations:** Department of Biological Sciences, Myongji University, Yongin 17058, Republic of Korea

**Keywords:** porins, OmpC, membrane integrity, cardiolipin, phospholipid transport, YhdP

## Abstract

Porins in the outer membrane (OM) of Gram-negative bacteria play two main functions: passage of various extracellular molecules and maintenance of membrane integrity. OmpC, a non-specific porin, is involved in both functions; however, the exact mechanism of maintenance of membrane integrity remains unknown. In this study, we found that inhibiting cardiolipin biosynthesis partially restored the growth defect of the *ompC* mutant under envelope stress. Among the three enzymes involved in cardiolipin biosynthesis, ClsABC, this effect is primarily associated with ClsA. Notably, the deletion of ClsA also suppressed the similar phenotypes of an *Escherichia coli* mutant lacking YhdP, a transmembrane protein involved in phospholipid transport from the inner membrane to the OM. Collectively, these results imply that OmpC may contribute to membrane integrity, partially through mechanisms linked to transport or biosynthesis of phospholipids such as cardiolipin.

## Introduction

The outer membrane (OM) of Gram-negative bacteria acts as a critical barrier, blocking the entry of toxic molecules into the periplasm. This impermeability primarily stems from the presence of lipopolysaccharide (LPS) in the outer leaflet of the OM (Mitchell and Silhavy, 2019). The saturated acyl chains of LPS restrict the passage of hydrophilic molecules, while the hydrophilic properties of the core and lipid A regions blocks hydrophobic molecules (Mitchell and Silhavy, 2019). Porins, which are β-barrel proteins in the OM, alleviate the impermeability of the OM by forming channels that allow small hydrophilic molecules, such as nutrients (Mitchell and Silhavy, 2019; Nikaido et al., 1983) and β-lactams (Nikaido et al., 1983).

In addition to facilitating transport, porins also contribute to membrane integrity (Choi and Lee, 2019). For instance, OmpA has a soluble periplasmic C-terminal domain that interacts with peptidoglycan and acts as a staple between the OM and peptidoglycan (Boags et al., 2019; Choi and Lee, 2019), similar to Braun's lipoproteins (Mandela et al., 2022). Therefore, the *ompA*(ΔC) mutant, defective in the soluble C-terminal domain, phenocopied the *ompA* mutant with sensitivity to various envelope stresses (Choi and Lee, 2019). Among non-specific porins, OmpA, OmpC, and OmpF, which are abundant in the OM, OmpA primarily contributes to the maintenance of membrane integrity, whereas OmpF mainly functions as a channel for small hydrophilic molecules, such as β-lactams (Choi and Lee, 2019). Notably, OmpC is important for both roles (Choi and Lee, 2019). Because OmpC and OmpF show high sequence (59% identity) and structural similarity (Baslé et al., 2006; Pauptit et al., 1991), OmpC can also function as a channel for small hydrophilic molecules, similar to OmpF. However, the molecular mechanisms by which OmpC maintains membrane integrity remain unknown. Because OmpC does not have a soluble periplasmic C-terminal domain, it does not function as OmpA.

In this study, we showed that the inactivation of OmpC, but not OmpF, increased sensitivity to various envelope stresses. Deletion of cardiolipin synthase ClsA partially restored the sensitivity of the *ompC* mutant to envelope stress. Deletion of ClsA also partially restored the sensitivity to envelope stress of the mutant defective in YhdP, a transport protein that plays a role in the transport of phospholipids from the inner membrane to the OM. These results imply that OmpC may be associated with phospholipid biosynthesis or transport.

## Materials and Methods

### Bacterial strains, plasmids, and culture conditions

All *E. coli* strains and plasmids used in this study are listed in Table S1. All primers used to construct the deletion strains and plasmids are listed in Table S2. All bacterial cells were cultured in Luria–Bertani (LB) medium at 37°C, unless otherwise mentioned. To prevent contamination by other bacterial cells, antibiotics, including ampicillin (100 μg/ml), chloramphenicol (5 μg/ml), tetracycline (10 μg/ml), and kanamycin (50 μg/ml), were added in the culture medium.

All *E. coli* mutant strains were constructed using λ red recombinase, as previously described (Datsenko and Wanner, 2000), with slight modifications. DNA products for deletion of the *clsA* gene were amplified by polymerase chain reaction (PCR) using primers with a 50 bp sequence for homologous recombination, as listed in Table S2. PCR products were purified using a PCR Purification Kit (Qiagen, USA). Purified PCR products were transformed into *ompC* mutant cells harboring the plasmid pKD46 expressing λ red recombinase by electroporation. Deletion mutants were selected in LB medium containing kanamycin. Deletion of the *clsA* gene was confirmed by PCR. To remove the kanamycin resistance gene inserted into the chromosome, the plasmid pCP20 expressing FLP recombinase was transformed into the mutant cells by electroporation, as previously described (Datsenko and Wanner, 2000). The plasmid pCP20 was cured by incubation at 37°C, instead of incubation at 42°C, for minimize physiological changes in the bacteria cells (Choi et al., 2024; Son et al., 2024). The other deletion mutants were constructed using the same method.

To construct the plasmid pACYC184-OmpC, a DNA fragment encompassing both the promoter region and open reading frame of OmpC (from -619 to +1239) was amplified by PCR using a forward primer possessing a synthetic BamHI site (underlined) (5'-CCCGTCCTGTGGATCCGGCAGTATAAAGGGTACAGT-3'), and a reverse primer possessing a synthetic EagI site (underlined) (5'-CCCAGCGCGTCGGCCGGCTGAAAACAATGAAAAAAG-3') (Table S2). After PCR purification, the DNA product was cloned into the plasmid pACYC184 digested with BamHI and EagI by infusion cloning (Clontech, USA), as reported previously (Choi et al., 2023). DNA insertion into the plasmid pACYC184 was confirmed by PCR using other primer sets (pACYC184-cfm-F and pACYC184-cfm-R) located within the plasmid pACYC184 (Table S2), and DNA sequencing.

### Measurement of bacterial growth under envelope stress

Overnight cultures of the indicated cells in LB medium were inoculated into fresh LB medium. When the OD_600_ reached approximately 0.8, the cells were serially diluted in fresh LB medium from 10^8^ to 10^4^ cells/ml in 10-fold steps. Aliquots of 2 μl from each dilution were spotted onto LB plates, LB plates containing indicated materials, or LB plates adjusted to pH 4.8 by adding sodium citrate (50 mM, final concentration) and adjusting the pH using 10 N HCl solution. After incubation at 37°C for 15–20 h, bacterial growth was photographed using a digital camera EOS 100D (Canon Inc., Japan). All bacterial growth data were obtained from three independent experiments.

### Transposon mutagenesis and identification of transposon insertion site

The *pir*-dependent transposon delivery vector pRL27 carrying a Tn5 transposase gene and a mini-Tn5 element encoding kanamycin resistance protein was used for random mutagenesis to identify mutants that suppress the EDTA sensitivity of the *ompC* mutant (Larsen et al., 2002). Plasmid pRL27 was amplified in *E. coli* DH5α*λpir* cells harboring the *pir* gene. Purified pRL27 plasmids were transformed into the *ompC* mutants via electroporation. The transformed cells were cultured in LB medium containing 2 mM ethylenediaminetetraacetic acid (EDTA) and kanamycin (50 μg/ml). As the plasmid pRL27 is not replicated in the *ompC* mutant, which does not carry the *pir* gene, the kanamycin resistance gene can be maintained only when chromosomal insertion of the mini-Tn5 element occurs. Among approximately 1,200 mutants containing chromosomal insertion of the mini-Tn5 element, 22 mutants that grew in LB medium containing 2 mM EDTA and kanamycin were obtained. After examining the growth of mutant cells under EDTA stress conditions, one mutant that most strongly suppressed the EDTA sensitivity of the *ompC* mutant was selected. To reveal the insertion site of the mini-Tn5 element, PCR was performed using a mini-Tn5 transposon inner primer (pRL27-inner-F1), 5'-GGTTGTAACACTGGCAGAGCATTACG-3, and an arbitrary primer (pRL27-SynArb1) consisting of a GGCGGT sequence and a random sequence (Table S2), as described previously (Lee et al., 2021). Genomic DNA from the suppressor strain was used as the template for PCR. After PCR purification, the PCR product was sequenced using another mimi-Tn5 transposon inner primer (pRL27-inner-F2), 5'-ATCAGCAACTTAAATAGCCTCTAAGG-3' (Table S2).

## Results

### Inactivation of OmpC porin results in increased sensitivity to various envelope stresses

**Fig. 1.**
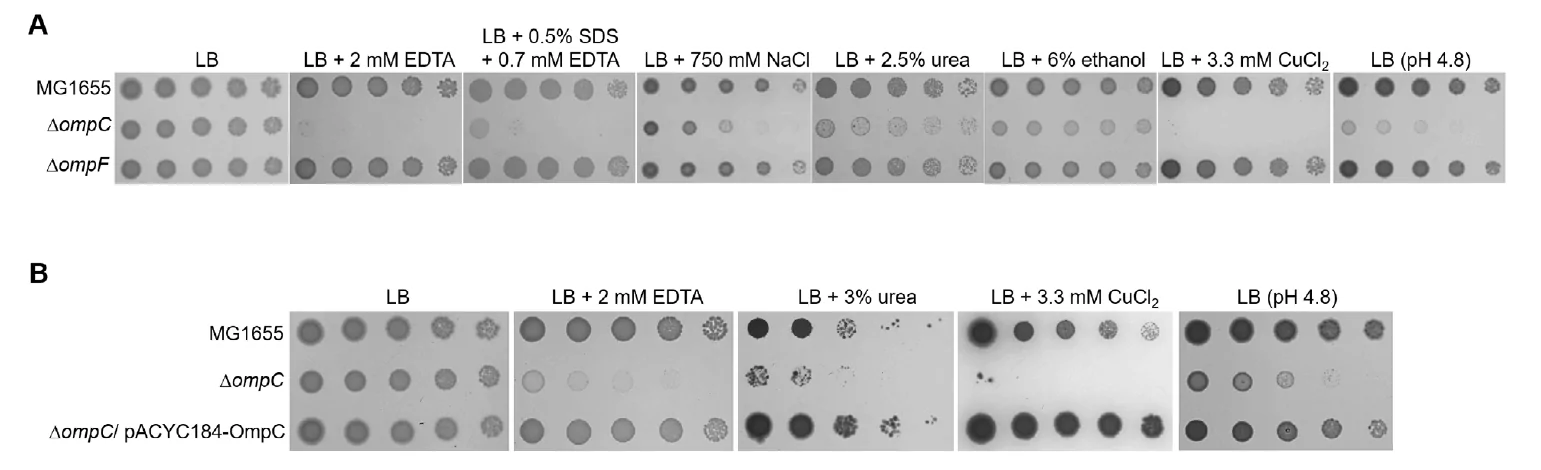
The sensitivity of the *ompC* mutant to various envelope stresses. (A) The effect of depletion of OmpC on overcoming envelope stresses. (B) Complementation of the sensitivity of the *ompC* mutant to envelope stresses. (A and B) The cells of the indicated strains were serially diluted from 10^8^ to 10^4^ cells/ml in 10-fold steps and spotted onto an LB plate, LB plates containing indicated materials, or an LB plate adjusted to pH 4.8. All experiments were performed in triplicate, and a representative image is presented.

Although OmpC and OmpF porins share 59% sequence identity and structural similarity (Baslé et al., 2006; Pauptit et al., 1991), they play distinct roles in maintaining membrane integrity (Choi and Lee, 2019). The *ompC* mutant exhibited heightened sensitivity to various envelope stresses, whereas the ompF mutant remained insensitive to these stresses (Fig. 1A). The phenotypes of the *ompC* mutant were almost completely complemented by the pACYC184 plasmid-based expression of the *ompC* gene (Fig. 1B). These results indicate that the OmpC porin is necessary for overcoming various envelope stresses.

### Defective cardiolipin biosynthesis partially suppresses the sensitivity of the *ompC* mutant to envelope stresses

**Fig. 2.**
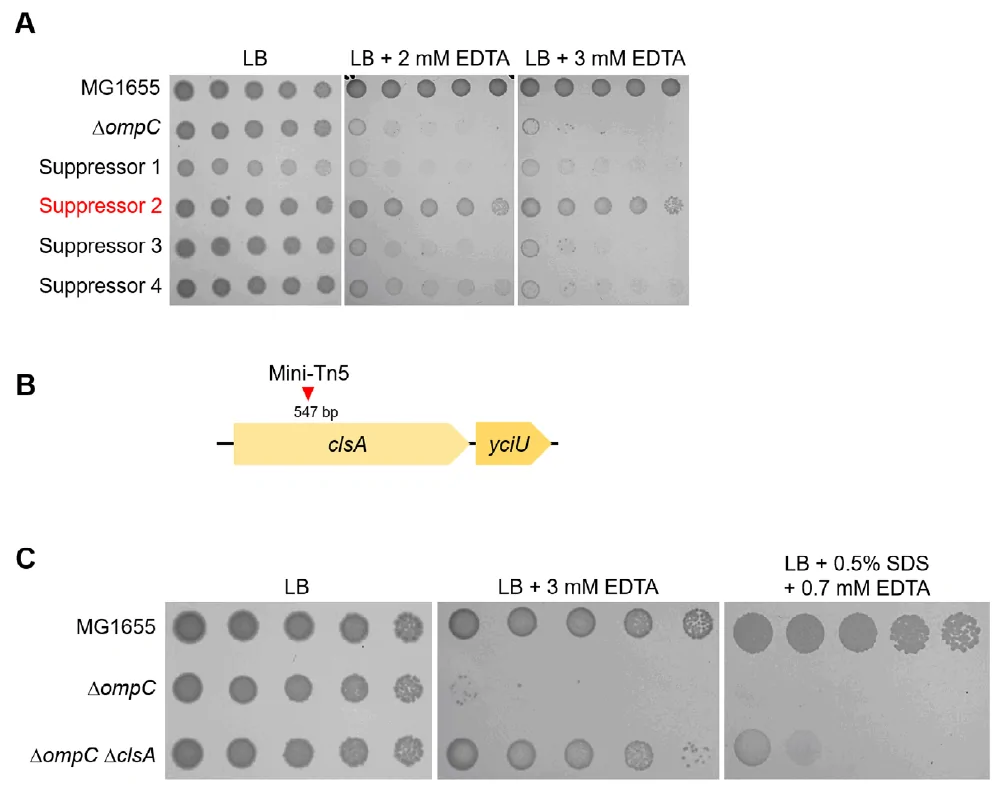
The effect of the cardiolipin synthase ClsA on the sensitivity of the *ompC* mutant to envelope stresses. (A) Isolation of the suppressor mutant of the *ompC* mutant. The cells of the indicated strains were serially diluted from 10^8^ to 10^4^ cells/ml in 10-fold steps and spotted onto LB plates with or without the indicated concentrations of EDTA. (B) Schematic representation of a mini-Tn5 insertion site. The mini-Tn5 insertion site of a suppressor mutant (suppressor 2) has been indicated using a red arrow. (C) The depletion of ClsA partially suppresses the sensitivity of the *ompC* mutant to envelope stresses. The cells of the indicated strains were serially diluted from 10^8^ to 10^4^ cells/ml in 10-fold steps and spotted onto LB plates with or without the indicated materials. The experiments were performed in triplicate, and a representative image is presented.

To analyze the molecular mechanism underlying the role of OmpC in the envelope stress response, we performed random transposon mutagenesis to identify suppressor mutations that restore the sensitivity of the *ompC* mutant to EDTA. Among the four suppressors isolated, only one suppressor (suppressor 2) restored the sensitivity of the *ompC* mutant to EDTA to a level similar to that of the wild-type strain (Fig. 2A). The transposon insertion was mapped within *clsA* (547 bp) encoding the major cardiolipin synthase (Nishijima et al., 1988; Tan et al., 2012) (Fig. 2B). To confirm the effect of the transposon insertion, we constructed an *ompC clsA* double mutant. Deletion of the *clsA* gene significantly restored the growth defect of the *ompC* mutant in the presence of EDTA (Fig. 2C). Additionally, deletion of the *clsA* gene partially restored the growth defect of the *ompC* mutant in the presence of SDS and EDTA (Fig. 2C). As a control, we tested the phenotype of the *clsA* mutant under these stress conditions. The *clsA* mutant was slightly sensitive to EDTA, whereas it was highly sensitive to SDS/EDTA (Fig. S1). Collectively, these results show that deletion of the *clsA* gene partially suppresses the sensitivity of the *ompC* mutant to envelope stress.

### The effect of other cardiolipin biosynthesis genes on the suppression is relatively weak

**Fig. 3.**
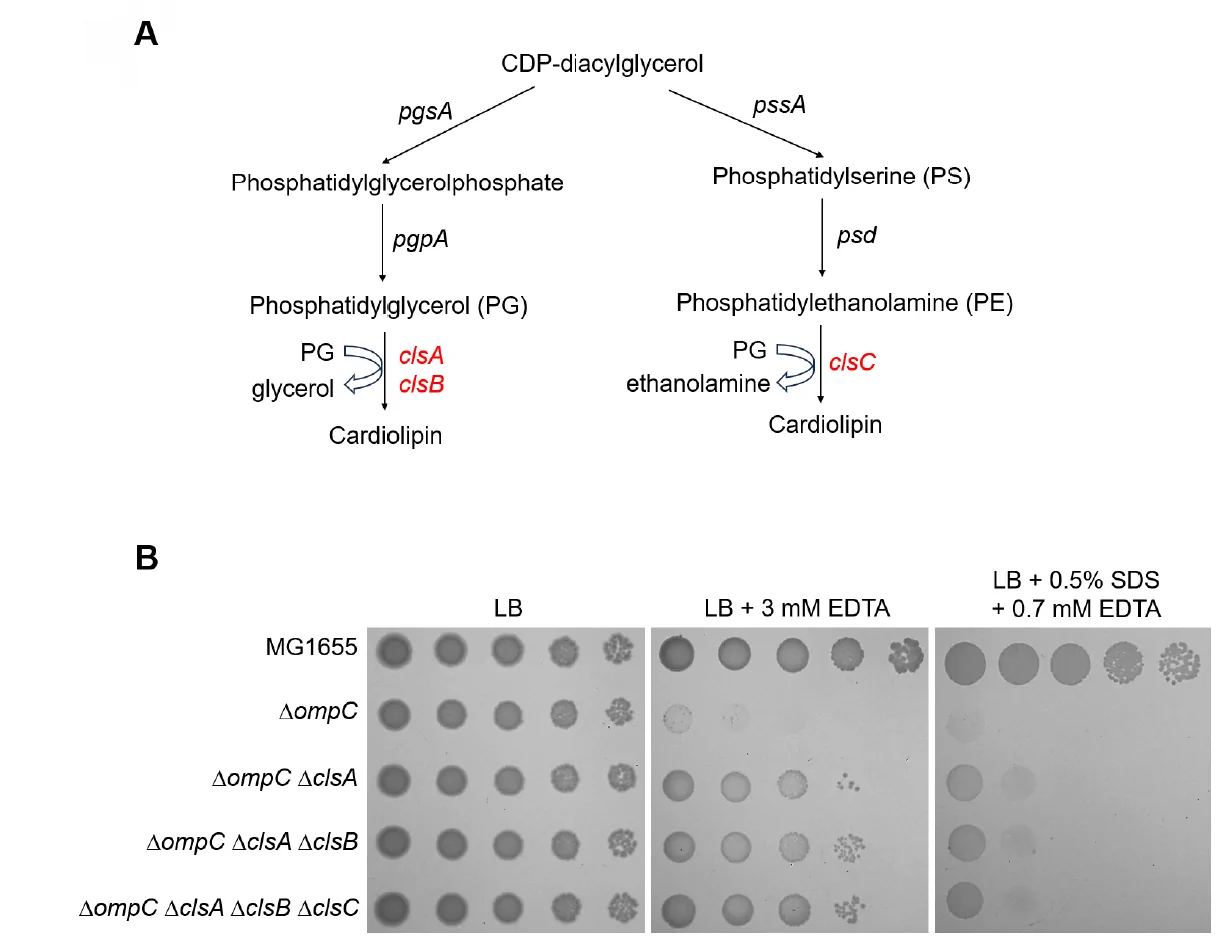
The effect of other cardiolipin synthases, ClsB and ClsC, on the sensitivity of the *ompC* mutant to envelope stresses. (A) Schematic representation depicting two distinct pathways of cardiolipin biosynthesis. ClsA and its isozyme ClsB synthesize cardiolipin through catalyzing the condensation of two PGs with a concomitant release of glycerol. ClsC synthesizes cardiolipin through catalyzing the condensation of PG and PE with a concomitant release of ethanolamine. (B) Additional depletions of ClsB and ClsC hardly affect suppression of the sensitivity of the *ompC* mutant to envelope stresses. The cells of the indicated strains were serially diluted from 10^8^ to 10^4^ cells/ml in 10-fold steps and spotted onto LB plates with or without the indicated materials. The experiments were performed in triplicate, and a representative image is presented.

Cardiolipin biosynthesis in *E. coli* occurs via two distinct pathways (Fig. 3A). ClsA and its isozyme, ClsB, which synthesizes cardiolipin, catalyze the condensation of two phosphatidylglycerols (PGs) with a concomitant release of glycerol (Nishijima et al., 1988), whereas ClsC synthesizes cardiolipin by catalyzing the condensation of PG and phosphatidylethanolamine (PE) with a concomitant release of ethanolamine (Tan et al., 2012). Among the three cardiolipin synthases, ClsA is the major enzyme in *E. coli*. ClsA contributes to cardiolipin synthesis during the exponential phase, whereas during the stationary phase, cardiolipin is synthesized through the combined activity of all three cardiolipin synthases (Nishijima et al., 1988; Pluschke et al., 1978; Shibuya et al., 1985; Tan et al., 2012). To determine the suppressive effects of ClsB and ClsC, we constructed *ompC clsA clsB* triple and *ompC clsA clsB clsC* quadruple mutants. Additional deletions of *clsB* and *clsC* did not affect the growth of the *ompC clsA* double mutant under envelope stress (Fig. 3B). Therefore, these results indicate that the major cardiolipin synthase, ClsA, plays an important role in the suppression of the phenotype of the *ompC* mutant.

### Defective cardiolipin biosynthesis also suppresses the sensitivity of the *yhdP* mutant to envelope stresses

**Fig. 4.**
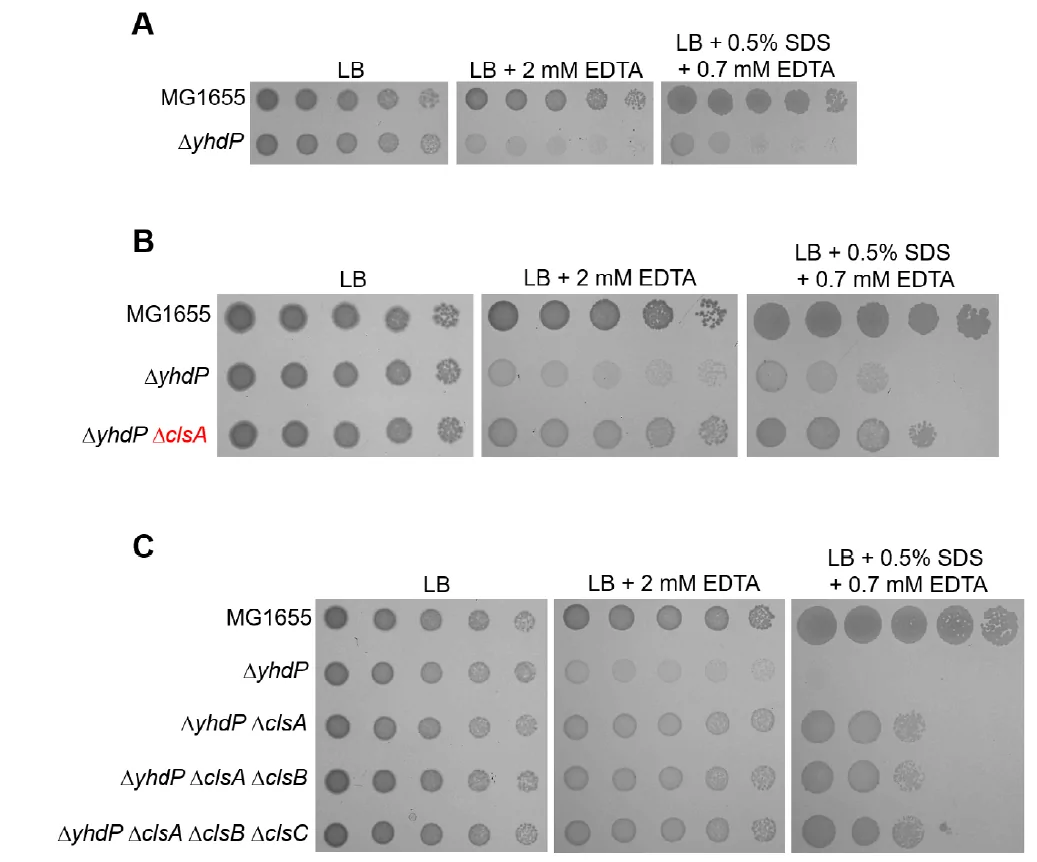
The effect of the cardiolipin synthase ClsA on the sensitivity of the *yhdP* mutant to envelope stresses. (A) The effect of depletion of YhdP on overcoming envelope stresses. (B) The depletion of ClsA partially suppresses the sensitivity of the *yhdP* mutant to envelope stresses. (C) Additional depletions of ClsB and ClsC hardly affect suppression of the sensitivity of the *yhdP* mutant to envelope stresses. (A–C) The cells of the indicated strains were serially diluted from 10^8^ to 10^4^ cells/ml in 10-fold steps and spotted onto LB plates with or without the indicated materials. The experiments were performed in triplicate, and a representative image is presented.

Phospholipid transporters, YhdP, TamB, and YdbH, which mediate phospholipid transport from the inner membrane to the OM, were recently identified (Douglass et al., 2022; Rai et al., 2024; Ruiz et al., 2021). Among these proteins, YhdP is essential for overcoming envelope stress (Sung et al., 2020), similar to OmpC. We investigated whether the depletion of cardiolipin biosynthesis suppressed the phenotype of the *yhdP* mutant. Similar to the *ompC* mutant, the *yhdP* mutant was sensitive to envelope stresses, such as EDTA and SDS/EDTA (Fig. 4A). Notably, the deletion of the *clsA* gene partially suppressed these phenotypes of the *yhdP* mutant (Fig. 4B). Additionally, similar to the *ompC* mutant, additional deletions of *clsB* and *clsC* had almost no effect on the growth of the *yhdP clsA* double mutant under envelope stress (Fig. 4C). Collectively, these results indicate that defects in cardiolipin biosynthesis partially suppress the sensitivity of the *yhdP* mutant to envelope stress, implying a genetic relationship between phospholipid transport and OmpC.

## Discussion

Porins in the OM not only function as channels for the penetration of environmental molecules, but also contribute to the mechanical stiffness of the bacterial envelope (Choi and Lee, 2019). OmpC plays an important role in both of these functions (Choi and Lee, 2019). Although the role of OmpC as a channel was well-established based on its structure (Nikaido, 2003; Nikaido et al., 1983; Pagès et al., 2008), the mechanism by which OmpC contributes to the stiffness of the bacterial envelope remains unknown. In this study, we provide genetic clues to the possibility that OmpC is associated with phospholipid biosynthesis or transport. The deletion of cardiolipin synthase suppressed the sensitivity of the *ompC* mutant to envelope stress (Fig. 2). Additionally, the deletion of cardiolipin synthase suppressed the sensitivity to envelope stress of the mutant defective in YhdP, a phospholipid transporter (Fig. 4). These results imply a genetic relationship between OmpC and phospholipid biosynthesis or transport.

The membrane of *E. coli* is composed of three phospholipids: PG, PE, and cardiolipin (DeChavigny et al., 1991; Killian et al., 1994; Pluschke et al., 1978). Enzymes involved in the biosynthesis of PG and PE, such as PgsA, PssA, PgpA, and Psd, are essential for bacterial growth (Goodall et al., 2018), whereas cardiolipin synthases ClsA, ClsB, and ClsC are not essential (Tan et al., 2012). In a random mutagenesis experiment using mini-Tn5, we identified suppressor mutants in which the mini-Tn5 transposon was inserted within the *clsA* gene (Fig. 2B), but did not identify any suppressor mutants in which the mini-Tn5 transposon was inserted within other genes involved in the biosynthesis of PG and PE. These results can be attributed to lethality upon disruption of essential genes involved in the biosynthesis of PG and PE rather than their irrelevance to suppression of the phenotype of the *ompC* mutant. Therefore, further experiments can be performed to investigate whether the decreased enzymatic activity of the enzymes involved in the biosynthesis of PG and PE suppresses the phenotype of the *ompC* mutant. Although three cardiolipin synthases, ClsA, ClsB, and ClsC, were present, only ClsA was associated with the suppression of the *yhdP* or *ompC* mutant phenotypes, and the effects of ClsB and ClsC were very weak (Figs. 3 and 4). These results suggest that ClsA is the main cardiolipin synthase. However, in this study, the cells at the exponential phase were inoculated in all experiments examining bacterial growth. Because ClsB and ClsC are expressed only during the stationary phase (Nishijima et al., 1988; Pluschke et al., 1978; Shibuya et al., 1985; Tan et al., 2012), further experiments are required to confirm the roles of ClsB and ClsC.

Several recent studies have identified phospholipid transporters involved in the transportation of phospholipids from the inner membrane to the OM (Douglass et al., 2022; Rai et al., 2024; Ruiz et al., 2021). Three proteins–YhdP, TamB, and YdbH–function as phospholipid transporters. Each protein is not essential, but the depletion of all three proteins leads to growth defects (Douglass et al., 2022; Ruiz et al., 2021). OM proteins are required for the transportation of phospholipids from the inner membrane to the OM. TamB cooperates with TamA, an OM protein with β-barrel structure, to transport phospholipids from the inner membrane to the OM (Tan and Chng, 2024). However, OM proteins that cooperate with YhdP and YdbH remain unidentified. In this study, we showed that decreased biosynthesis of phospholipids partially restored the phenotypes of the *yhdP* and *ompC* mutants (Figs. 2 and 4), implying that OmpC may participate in the transport of phospholipids from the inner membrane to the OM. However, this assumption needs to be confirmed by further experiments.

Our results showed that each deletion mutant of *yhdP* and *clsA* did not grow under SDS/EDTA stress conditions, whereas the *yhdP clsA* double mutant partially restored the growth under SDS/EDTA stress conditions (Figs. 4 and S1). Notably, several reports showed that cardiolipin facilitates the transportation of LPS in the inner membrane into the OM (Douglass et al., 2021; Gorzelak et al., 2021). Because the OM is an asymmetric membrane composed of phospholipid and LPS, coordinated transport of phospholipid and LPS is necessary for its stiffness. Therefore, our results can be explained based on coordinated transport of phospholipid and LPS. In other words, impaired transport of either phospholipid or LPS induces the OM integrity defect, whereas simultaneously impaired transport of both phospholipid and LPS could partially restore the OM integrity. Therefore, further experiments are required to determine whether the effect of ClsA depletion on the *yhdP* or *ompC* mutant is mediated by directly decreased cardiolipin biosynthesis or indirectly impaired LPS transport.

## Supplementary Information

Fig. S1. The effect of the cardiolipin synthase ClsA on the sensitivity to envelope stresses. The cells of the indicated strains were serially diluted from 10^8^ to 10^4^ cells/ml in 10-fold steps and spotted onto LB plates with or without the indicated materials. The experiments were performed in triplicate, and a representative image is presented.

Table S1. *Escherichia coli* strains and plasmids used in this study

Table S2. Oligonucleotides used in this study
