# Supplementary material for: Inhibition of cardiolipin biosynthesis partially suppresses the sensitivity of an Escherichia coli mutant lacking OmpC to envelope stress
Source: J Microbiol. 2025 Nov 30;63(11):e2507004. doi: 10.71150/jm.2507004 (PMC13577075; doi:10.71150/jm.2507004)
Supplement: Supplementary file 1 [file jm-2507004-Supplementary-Fig-S1.pdf]

Supplementary materials

Supplementary Figure

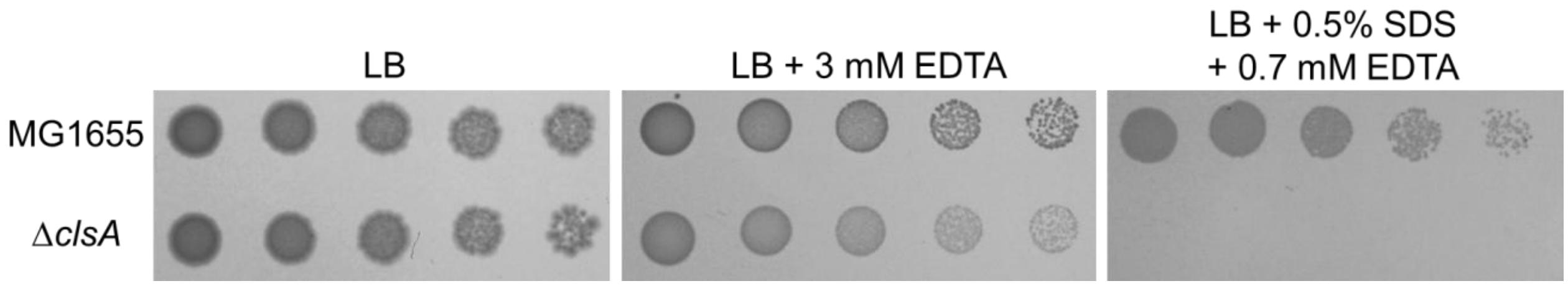

Fig. S1. The effect of the cardiolipin synthase ClsA on the sensitivity to envelope stresses. The cells of the indicated strains were serially diluted from 10^8 to 10^4 cells/ml in 10-fold steps and spotted onto LB plates with or without the indicated materials. The experiments were performed in triplicate, and a representative image is presented.
