# Supplementary material for: Inhibition of cardiolipin biosynthesis partially suppresses the sensitivity of an Escherichia coli mutant lacking OmpC to envelope stress
Source: J Microbiol. 2025 Nov 30;63(11):e2507004. doi: 10.71150/jm.2507004 (PMC13577075; doi:10.71150/jm.2507004)
Supplement: Supplementary file 2 [file jm-2507004-Supplementary-Table-S1.pdf]

Supplementary Tables

Table S1. *Escherichia coli* strains and plasmids used in this study

Strain or plasmid	Genotype or phenotype	Source or reference
**Strains**		
MG1655	F^- λ^- *ilvG*$^-$ *rfb*-50 *rph*-1. Wild type *E. coli* K-12	Blattner et al. (1997)
MG1655 Δ*ompC*	MG1655 *ompC::frt*	Choi and Lee (2019)
MG1655 Δ*ompF*	MG1655 *ompF::frt*	Choi and Lee (2019)
MG1655 Δ*yhdP*	MG1655 *yhdP::frt*	Sung et al. (2020)
MG1655 Δ*clsA*	MG1655 *clsA::frt*	This study
MG1655 Δ*ompC* Δ*clsA*	MG1655 *ompC::frt clsA::frt*	This study
MG1655 Δ*ompC* Δ*clsA* Δ*clsB*	MG1655 *ompC::frt clsA::frt clsB::frt*	This study
MG1655 Δ*ompC* Δ*clsA* Δ*clsB* Δ*clsC*	MG1655 *ompC::frt clsA::frt clsB::frt clsC::frt*	This study
MG1655 Δ*yhdP* Δ*clsA*	MG1655 *yhdP::frt clsA::frt*	This study
MG1655 Δ*yhdP* Δ*clsA* Δ*clsB*	MG1655 *yhdP::frt clsA::frt clsB::frt*	This study
MG1655 Δ*yhdP* Δ*clsA* Δ*clsB* Δ*clsC*	MG1655 *yhdP::frt clsA::frt clsB::frt clsC::frt*	This study
**Plasmids**		
pACYC184	A low copy number cloning vector; Cm^R, Tet^R	Schottel et al. (1981)
pACYC184-OmpC	pACYC184-based expression vector for OmpC, Cm^R	This study
pKD13	Template plasmid for the amplification of the kanamycin-resistance gene bordered by FRT sites, Km^R	Datsenko and Wanner (2000)

pKD46	λ Red recombinase expression plasmid Ts replicon, Amp^R	Datsenko and Wanner (2000)
pCP20	FLP helper plasmid Ts replicon, Amp^R, Cm^R	Datsenko and Wanner (2000)
pRL27	Tn5-RL27 (Km^R-*ori*R6 K) delivery vector: circularized PCR fragment from pRL23 (primers *tet*Ap-for and *oriT-rev*)	Larsen et al. (2002)

Supplementary references

Blattner FR, Plunkett G 3rd, Bloch CA, Perna NT, Burland V, et al. 1997. The complete genome sequence of *Escherichia coli* K-12. *Science.* 277: 1453–1462.

Choi U, Lee CR. 2019. Distinct roles of outer membrane porins in antibiotic resistance and membrane integrity in *Escherichia coli*. *Front Microbiol.* 10: 953.

Datsenko KA, Wanner BL. 2000. One-step inactivation of chromosomal genes in *Escherichia coli* K-12 using PCR products. *Proc Natl Acad Sci USA.* 97: 6640–6645.

Larsen RA, Wilson MM, Guss AM, Metcalf WW. 2002. Genetic analysis of pigment biosynthesis in *Xanthobacter autotrophicus* Py2 using a new, highly efficient transposon mutagenesis system that is functional in a wide variety of bacteria. *Arch Microbiol.* 178: 193–201.

Schottel JL, Bibb MJ, Cohen SN. 1981. Cloning and expression in *Streptomyces lividans* of antibiotic resistance genes derived from *Escherichia coli*. *J Bacteriol.* 146: 360–368.

Sung CG, Choi U, Lee CR. 2020. Phenotypic characterization of a conserved inner membrane protein YhcB in *Escherichia coli*. *J Microbiol.* 58: 598–605.
