# Supplementary material for: Inhibition of cardiolipin biosynthesis partially suppresses the sensitivity of an Escherichia coli mutant lacking OmpC to envelope stress
Source: J Microbiol. 2025 Nov 30;63(11):e2507004. doi: 10.71150/jm.2507004 (PMC13577075; doi:10.71150/jm.2507004)
Supplement: Supplementary file 3 [file jm-2507004-Supplementary-Table-S2.pdf]

Table S2. Oligonucleotides used in this study

Name	Oligonucleotide sequence (5'–3')	Use(s)
ClsA-FRT-F	TCCATATGCGTAAACTCATAACAATGCGCTTTCAAAAGGATTTCTAATCTGTGTAGGCTGGAGCTGCTTC	Deletion
ClsA-FRT-R	CGTAAAAACACACGCATCAGATTGACCTTTAAGGCTTCGACCACTTCAATATTCCGGGGATCCGTCGACC	
ClsB-FRT-F	ACATGGCGACACCTTTCTGATCATGCCCCTTTAAGTGCGGAGATTCATTTGTGTAGGCTGGAGCTGCTTC	
ClsB-FRT-R	GTGAGCATTTTCAAATAATGGCGTTCAATATCATCGCGATGTTCTTCGTTATTCCGGGGATCCGTCGACC	
ClsC-FRT-F	AAACFCCCACCTCTACGAAAGACTCCTTACCCAACAAGGAGATGAATGATGTGTAGGCTGGAGCTGCTTC	
ClsC-FRT-R	GCATCGTTATGCCAGGAGGCGGGTAACTCGATGCGATCCGCCATTTCACCATTCCGGGGATCCGTCGACC	
ClsA-cfm-F	TGCTTTTCCCGCGTTTTTATTTGCCAGATG	Deletion confirm
ClsA-cfm-R	TTTCGCGGAAGGATAATACTGACATCCACC	
ClsB-cfm-F	GCCAGCGCGCCAACCGCGTTGCCGCTGCGG	
ClsB-cfm-R	GAAGTAGGCGTTGGCGATAATCACTTCCCG	
ClsC-cfm-F	ACAGGTATACTTTGTCTGTTATGATGAAGA	
ClsC-cfm-R	CAATGTTCCATCAACCAGATGATTTATAAA	
pACYC184-OmpC-F	CCCGTCCTGTGGATCCGGCAGTATAAAGGGTACAGT	pACYC184 cloning
pACYC184-OmpC-R	CCCAGCGCGTCGGCCGGCTGAAAACAATGAAAAAAG	

pACYC184-cfm-F	CTTGGAGCCACTATCGACTACGCGATCATG	
pACYC184-cfm-R	CCGCCGGAAGCGAGAAGAATCATAATGGG G	
pRL27-inner-F1	GGTTGTAACACTGGCAGAGCATTACG	
pRL27-SynArb1	CCCACAGGAAGGTATTCTGGAAGATACGGC GGT	Random mutagenesis
pRL27-inner-F2	ATCAGCAACTTAAATAGCCTCTAAGG	
